# Structured succession of a cross-kingdom microbiome drives degradation and detoxification of oolong tea waste

**DOI:** 10.3389/frmbi.2026.1871476

**Published:** 2026-09-24

**Authors:** Guanming Ye, Xiaoqing Yu, Marsena Jasiel Ismaiah, Ren Ke, Ziheng Zeng, Jiarui Wang, Shuning Lan, Han Yu, Huiyu Liu, Shanyu Xie, Kin Sum Leung, Lu Zhang, Jetty Chung-Yung Lee, Olivier Habimana

**Affiliations:** 1 School of Biological Sciences, The University of Hong Kong, Hong Kong, Pokfulam, China; 2 Biotechnology and Food Engineering Program; Guangdong Technion-Israel Institute of Technology, Shantou, China

**Keywords:** aerobic degradation, antibiotic resistance genes, circular economy, metagenomics, microbial succession, oolong tea waste

## Abstract

The spontaneous aerobic degradation of oolong tea waste offers a promising but underexplored route for sustainable waste valorization. In this study, we used whole-genome metagenomic sequencing to examine microbial succession and functional gene dynamics over a 35-day degradation period. Our results uncovered a three-phase ecological progression: an initial mesophilic phase (Days 0–7) dominated by Pseudomonadota, which reached 93.4% relative abundance and specialized in simple substrate utilization; a thermotolerant transition (Days 8-15, 30-33°C) characterized by increased abundance of glycoside hydrolases (GH5, GH13); and a maturation phase (Days 16–35) marked by the emergence of lignocellulose-degrading taxa such as *Cellulomonas* and *Microbacterium*, accompanied by a 78% reduction in antibiotic resistance genes. Profiling of carbohydrate-active enzymes revealed a sequential shift from pectinases (GH28) to hemicellulases (GH43), while mobile genetic elements declined substantially during the maturation phase. These results indicate that tea waste degradation follows structured microbial succession patterns influenced by abiotic factors and suggests a potential self-purification capacity through the attenuation of antibiotic resistance genes. This study establishes a foundation for optimizing composting processes and designing targeted bioaugmentation strategies to enhance tea waste valorization.

## 1 Introduction

Tea intake generates considerable biological waste challenges in the form of organic waste comprising discarded leaves, stems, and dust (Duarah et al., 2023). Globally, tea production generates approximately 1.5–2.0 million metric tons of waste annually, with major producers such as China, India, and Kenya each contributing hundreds of thousands of tons per year (Sarneel et al., 2024). This residue accumulates yearly, stressing landfills and increasing greenhouse gas emissions. The waste generated from oolong tea production, characterized by an intricate matrix of lignin, cellulose, and polyphenols, offers a promising yet largely uncharted opportunity for sustainable valorization (Çakmak et al., 2024; Debnath et al., 2021; Duarah et al., 2023; Seth et al., 2023). Conventional waste management techniques like composting and anaerobic decomposition frequently fail to fully utilize this asset’s biochemical potential (Nayak et al., 2024, Wang et al., 2023). A deeper understanding of the inherent microbial processes driving its decomposition is therefore critical for developing efficient bioconversion strategies within a circular economy framework.

Current research on tea waste has primarily focused on chemical composition or employed pre-inoculated fermentation systems for targeted enzyme production (Lin et al., 2022; Ma et al., 2017). Studies have documented specific polyphenol degradation pathways or the bioconversion of tea residues into biofuels using engineered microbial consortia (Ma et al., 2017). However, the inherent microbial succession during uncontrolled aerobic decomposition, particularly the temporal taxonomic and functional shifts within both bacterial and fungal communities that correlate with physicochemical changes, has not been systematically investigated (Yang et al., 2023; Zhao et al., 2022). Unlike prior studies that employed pre-inoculated fermentation or chemical characterization alone, our metagenomic approach captures the natural, unamended succession across the entire degradation timeline. This knowledge gap limits our ability to harness natural microbial consortia for efficient waste processing or to design informed bioaugmentation strategies that reflect the true complexity of the decomposition process.

A 35-day degradation period was selected for this study, as prior composting studies have shown that active degradation of tea waste typically concludes within 28–35 days (Fan et al., 2017; Zhang et al., 2022), providing a suitable window to capture the complete successional trajectory. We propose that aerobic decomposition of oolong tea byproducts follows a predictable, phase-dependent sequence determined by abiotic variables including pH, temperature, and oxygen availability (Yu et al., 2007; Zhang et al., 2022). This succession will involve distinct phases of multi-kingdom microbial activity. In the initial mesophilic phase (Days 0–7), we anticipate dominance by fast-growing bacterial generalists (e.g., Pseudomonadota) capable of metabolizing simple sugars and polyphenols. As labile substrates become depleted, we predict a thermotolerant transition (Days 8–15), with temperatures rising to 30-33°C. This temperature range is consistent with self-heating observed during aerobic decomposition of plant-based agricultural residues with similar lignocellulosic composition (Fan et al., 2017; Yu et al., 2007), where heat generation from exothermic microbial metabolism is moderated by the ambient laboratory conditions and the relatively small reactor scale compared to industrial composting piles. Similar phase-dependent successions have been observed in the aerobic degradation of other lignocellulosic agricultural residues such as rice straw, wheat straw, and green waste, where mesophilic colonizers are progressively replaced by thermotolerant and then specialized lignocellulolytic taxa as labile substrates are depleted (Yu et al., 2007; Zhang et al., 2022). This phase should culminate in a maturation phase (Days 16–35) dominated by specialized bacterial lignocellulolytic taxa, characterized by functional redundancy, humification, and significant attenuation of antibiotic resistance genes (ARGs) and virulence factors, indicative of system self-purification.

Here, deterministic succession refers to a non-random community assembly under environmental filtering and substrate availability rather than stochastic neutral processes. The self-purification capacity refers to the inherent potential of decomposer community composition in degrading mobile genetic elements and their associated antibiotic resistance genes during compost maturation, which may help on reducing biosafety risks from compost application.

## 2 Materials and methods

### 2.1 Experimental setup and sample preparation

Oolong tea waste was collected from a local tea processing facility and consisted of a homogenized mixture of discarded leaves, stems, and dust from four commercially available varieties, specifically two Phoenix Dancong and two Ya Shi Xiang teas. Upon arrival at the laboratory, the composite waste was air-dried at approximately 22°C for 48 hours to reduce the initial moisture content and facilitate homogenization (**Supplementary Figure S1**). To standardize the substrate and simulate industrial tea extraction, 100 g of the blended tea waste was infused in boiling ultrapure water at a ratio of 1:20 (w/v) for five minutes using 316-grade stainless steel cookware. The brewed leaves were carefully separated, permitted to cool to approximately 25°C ( ± 2°C), and subsequently stored at 4°C for the degradation experiment. A representative composite sample was created by combining equal weights (approximately 500 g each) of material from each variety and mixed thoroughly using a mechanical blender to ensure homogeneity. At no point in the experiment was any external microbial culture introduced; the decomposition process relied solely on the native microbial consortium present within the fresh tea dregs.

Aerobic degradation was carried out in three replicate insulated polypropylene reactors (600 × 400 × 320 mm) constructed from food-grade material to minimize contamination. Each reactor contained 1.9 kg (dry weight) of the prepared tea waste substrate and was maintained under controlled laboratory conditions at an ambient temperature of 22°C. The reactors were insulated with a 2.5 cm thick layer of polystyrene foam to maintain a stable internal temperature. Aeration was supplied through 16 evenly spaced 25 mm holes on each reactor’s top surface. The substrate was mixed three times daily to ensure oxygen and moisture distribution.

Moisture content (gravimetric water content, i.e., water mass per wet mass) was maintained at approximately 90% through periodic addition of sterile water, as this level has been shown to optimize microbial activity in lignocellulosic degradation systems (Liang et al., 2003; Richard et al., 2002). Moisture was monitored using a calibrated digital soil tester (LY-201, Jiangrentang, China), with measurements taken at multiple locations within each reactor to ensure accuracy.

The 35-day duration was selected based on prior composting studies showing that active degradation of tea waste typically concludes within 28–35 days (Fan et al., 2017; Zhang et al., 2022). Decomposition rate was monitored by measuring dry weight loss at each sampling time point, with samples dried at 105°C for 24 hours to constant weight.

To prevent cross-contamination between replicate reactors, dedicated sterile paddles and spatulas were used for each reactor, with operators changing gloves and cleaning work surfaces with 70% ethanol between handling different reactors. To monitor for cross-contamination, sterile swabs were rubbed across workbench areas, and operator gloves at each sampling time point, then inoculated onto LB agar and incubated for 48 hours at 30°C. No growth was observed (data not shown), confirming that cross-contamination of samples was avoided.

### 2.2 Physicochemical monitoring

To monitor vital environmental parameters affecting biological and chemical processes, daily assessments of temperature, pH, and moisture were conducted at three time points (approximately 6-hour intervals: 08:00, 14:00, 20:00) to ensure thorough observation of ecosystem changes. Temperature measurements were made on a three time intervals (08:00, 14:00 and 20:00) during the day to account for diurnal variation. The 14:00 readings consistently showed the highest temperatures, representing the internal core temperature of the biomass, while the 08:00 and 20:00 readings reflected periods of heat dissipation following mixing. The mixing procedure, performed three times daily, temporarily reduced internal temperatures by approximately 2-3°C through aeration and heat loss, consistent with standard composting practice (Yu et al., 2007). Temperature was recorded via a digital thermometer inserted into the compost substrate’s core. For pH measurement, a 1:10 (w/v) suspension of each compost sample in aseptic distilled water was prepared. The slurry underwent continuous agitation for 30 minutes, followed by a 10-minute settling period before pH measurement of the supernatant using a calibrated digital pH meter. Key physicochemical dynamics, including temperature peaks (30–33°C) and pH fluctuations (initial 6.2 → minimum 5.9 → stabilization at 6.7–6.8), were correlated with microbial succession data. All instruments were calibrated daily according to manufacturer specifications using certified standards.

### 2.3 DNA extraction and sequencing

Genomic deoxyribonucleic acid (gDNA) was isolated from samples gathered at six different time points (Days 0, 3, 7, 14, 28, 35) utilizing the ZymoBIOMICS DNA Miniprep Kit (D4300), in rigorous compliance with the manufacturer’s guidelines. The Day 0 samples were obtained immediately after homogenization and reactor loading, before the degradation process started. Approximately 0.25 g of compost material was used for each extraction. The gDNA was eluted in 50 µL buffer and stored at -20°C for subsequent analysis. DNA quality was assessed via Nanodrop spectrophotometry (Thermo Scientific NanoDrop 2000) to determine A260/280 and A260/230 ratios, with targets of >1.8 and >1.7, respectively. DNA concentration was assessed with a Qubit 4.0 Fluorometer and the Qubit dsDNA HS Assay Kit.

Whole-genome metagenomic sequencing was performed on the NovaSeq 6000 platform (Novogene, Shenzhen) using 350 bp insert libraries and PE150 paired-end reads, generating approximately 20 Gb of raw data per sample. This sequencing depth was chosen to ensure sufficient coverage for comprehensive metagenomic assembly, consistent with recommended depths for complex microbial communities (Quince et al., 2017). Library preparation was conducted using the Illumina TruSeq DNA PCR-Free Library Preparation Kit. Adapter-trimmed and quality-filtered reads were assembled using MEGAHIT (v1.2.9) with a k-mer range of 21–121.

### 2.4 Bioinformatic analysis

Taxonomic profiling was conducted by aligning quality-filtered reads to the MicroNR database using DIAMOND (v2.0.4) with default parameters (E-value cutoff 1e-5). Taxonomic abundance was determined from alignment results, with relative abundances calculated at kingdom and phylum levels based on the Unigenes database generated through the MicroNR annotation pipeline. Shannon, ACE, and Chao1 alpha diversity indices were estimated via QIIME2(v2022. 2). Beta diversity was estimated by PCoA with Bray-Curtis dissimilarity matrices calculated from relative abundance data across samples.

Functional annotation of genes was performed through alignment to specialized databases. Carbohydrate-active enzymes (CAZymes) were annotated using dbCAN4 (v4.0.0) against the CAZy database with HMMER scans (E-value cutoff 1e-5). Antibiotic resistance genes (ARGs) were identified using the Comprehensive Antibiotic Resistance Database (CARD; v3.2.6) with the Resistance Gene Identifier (RGI) using default parameters. The identified virulence and pathogenicity genes were characterized using the Pathogen-Host Interactions (PHI) database. Metabolic pathways were identified through KEGG database annotation.

Core-pan genome analysis was performed using Roary (v3.13.0) with default parameters to identify the core (genes present in ≥99% of genomes) and pan (genes present in <99% of genomes) genome across all time points. This analysis was used to assess genetic diversity and specialization of the microbial community over time.

Statistical validation of community differences across time points was performed using PERMANOVA (ADONIS) with 999 permutations, confirming significant separation between degradation phases (p < 0.001). All visualization outputs including heatmaps, alpha diversity boxplots, and PCoA plots were generated from the standard Novogene analysis pipeline as detailed in their documentation.

## 3 Results

### 3.1 A three-phase microbial succession dictates aerobic degradation dynamics

Metagenomic analysis of the spontaneous aerobic degradation of oolong tea waste revealed a highly structured, deterministic succession of microbial communities, consistent with deterministic assembly processes, over the 35-day process (**Figure 1**). This progression occurred through three distinct ecological phases, mesophilic colonization, thermotolerant transition, and maturation, each characterized by unique taxonomic compositions and functional profiles tightly linked to evolving substrate availability.

**Figure 1:**
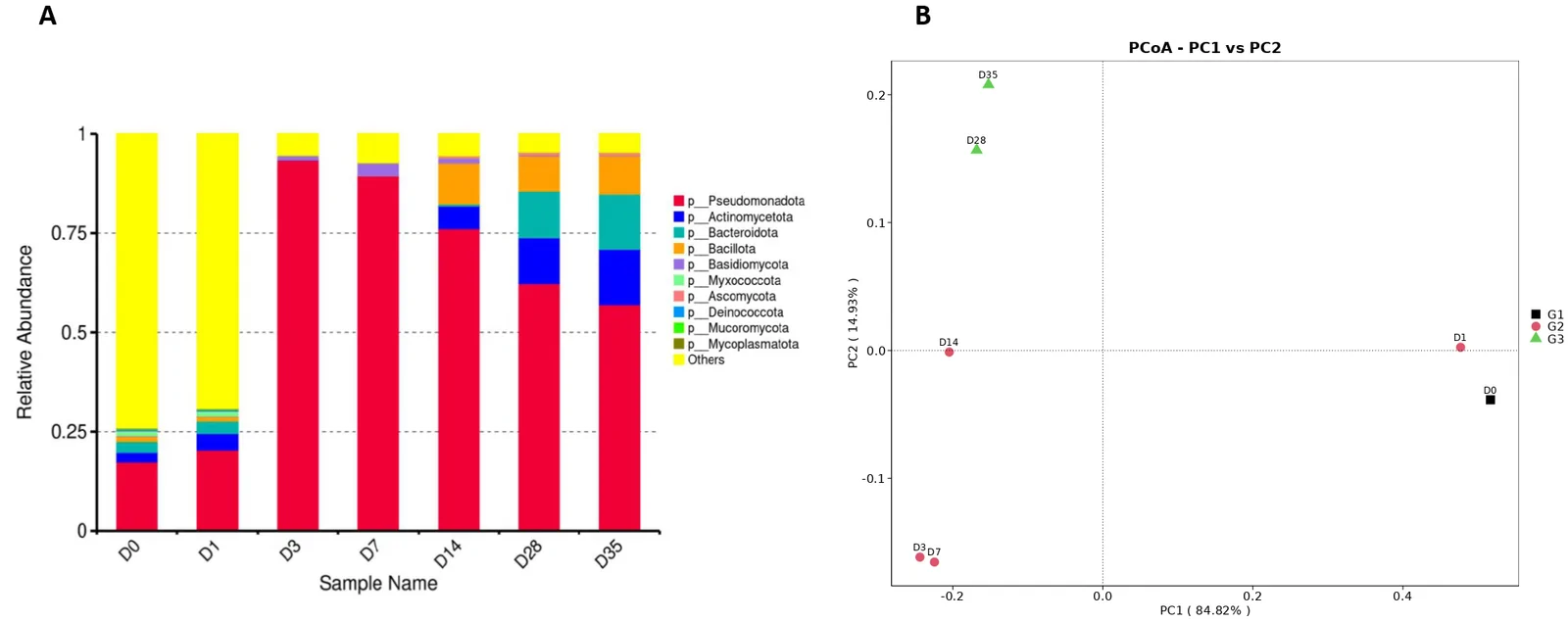
Microbial community succession during aerobic degradation. **(A)** Stacked bar chart showing the relative abundance of dominant bacterial phyla and the fungal phylum Ascomycota across six time points (D0 to D35; D = day). **(B)** PCoA plot based on Bray-Curtis dissimilarity, illustrating the significant temporal shift in community structure and the clustering of samples by degradation phase (I, II, III). Percentages indicate the variance explained by each principal coordinate.

### 3.2 Phase I: mesophilic colonization and rapid substrate depletion

The initial phase (Days 0–7) was marked by the rapid proliferation of Pseudomonadota, which surged from 17.4% to a peak relative abundance of 93.4% by Day 3 (**Figure 1A**). This bacterial dominance was accompanied by a significant contraction in overall microbial diversity, as evidenced by a decline in alpha diversity (**Table 1**), a trend further visualized in the phase-aggregated alpha diversity analysis (**Supplementary Figure S2**). Concurrently, the relative abundance of the fungal phylum Ascomycota was detected at a low level (≤ 0.5%) during this early phase. The microbial consortium was chiefly constituted of rapidly proliferating, versatile genera within the *Enterobacter*, *Pantoea*, and *Erwinia* assemblages (**Supplementary Figure S3**), which are proficient at catabolizing monosaccharides and organic acids prevalent in newly processed tea waste (Vilanova et al., 2015). A key phenotypic indicator of this metabolic burst was the production of extracellular polymeric substances, manifesting as a viscous mucus coating on the tea leaves, suggestive of biofilm formation for enhanced substrate adhesion and cooperative degradation. This observation aligns with recent findings on biofilm development during organic waste degradation (McKew et al., 2013; Yan et al., 2024), where EPS production and biofilm maturation enhance enzyme retention and create microenvironments that facilitate breakdown of recalcitrant compounds.

**Table 1:** Alpha diversity indices of microbial communities across the 35-day aerobic degradation of oolong tea waste.

Sample	ACE	Chao1	Shannon	Simpson	Observed_species	Goods_coverage
D0	4071.83	4144.12	3.95	0.94	3972.00	1.00
D1	4326.28	4363.32	4.13	0.95	4295.00	1.00
D3	1383.13	1372.03	3.61	0.88	1309.00	1.00
D7	2088.70	2122.01	3.67	0.89	1915.00	1.00
D14	5720.89	5771.89	4.32	0.93	5625.00	1.00
D28	8013.35	8061.98	5.08	0.97	7925.00	1.00
D35	8430.87	8511.23	5.23	0.98	8361.00	1.00

Functional profiling confirmed this community was primed for the utilization of labile compounds, exhibiting high activity in pathways for glycolysis and the pentose phosphate cycle. This was complemented by a high prevalence of genes encoding pectinases (GH28) and auxiliary activities (AA1) (**Figure 2**), facilitating the primary breakdown of pectin-rich plant cell walls.

**Figure 2:**
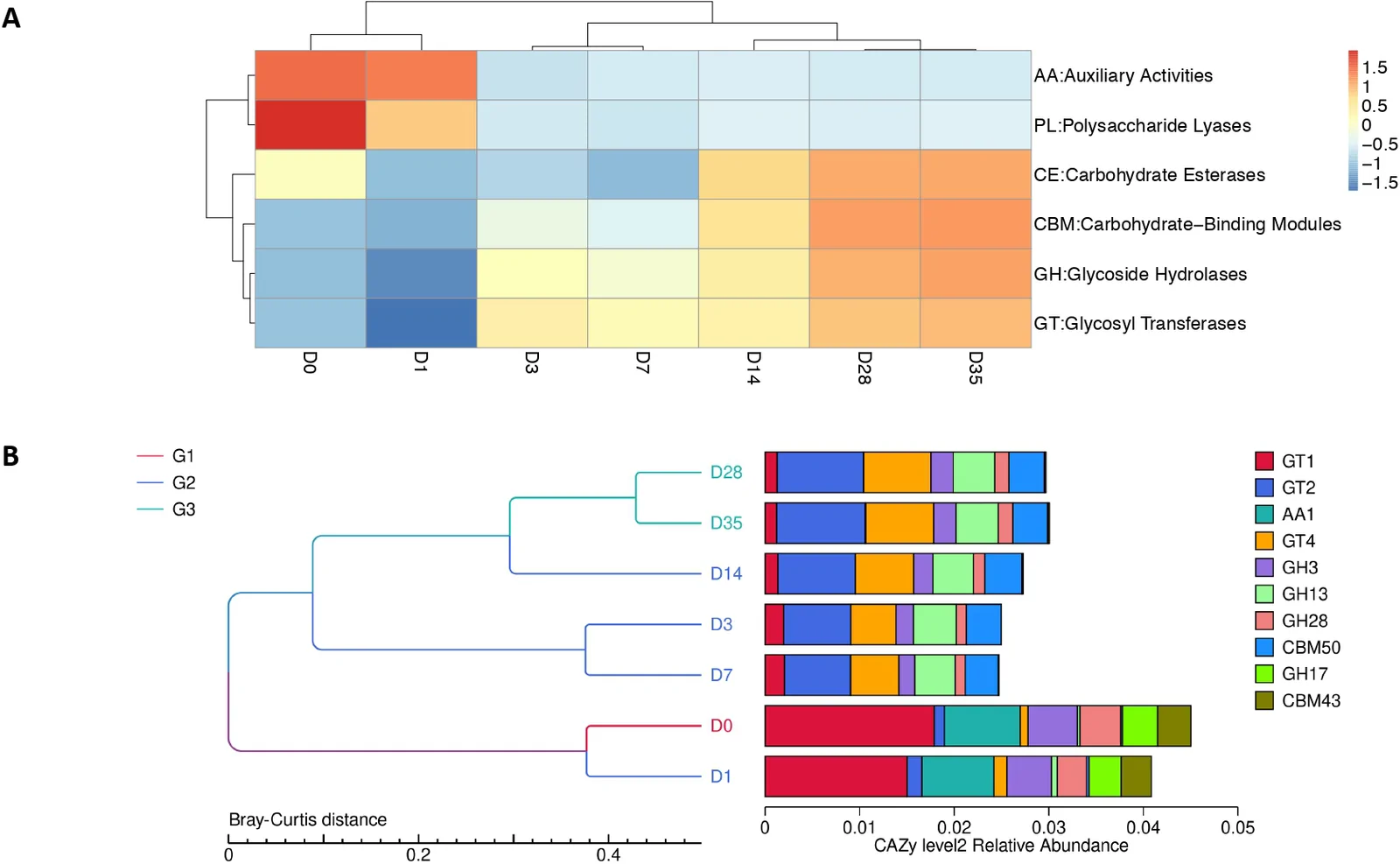
Dynamics of carbohydrate-active enzymes (CAZymes). **(A)** Heatmap showing the relative abundance of key CAZy families (e.g., GH28, GH5, GH13, GH43, CBM48) over time, demonstrating a functional succession from pectinolytic to cellulolytic and hemicellulolytic activities. **(B)** Bar chart displaying the relative abundance of level 1 CAZy categories (AA, Auxiliary Activities; CBM, Carbohydrate-Binding Modules; CE, Carbohydrate Esterases; GH, Glycoside Hydrolases; GT, Glycosyl Transferases; PL, Polysaccharide Lyases) throughout the degradation process.

### 3.3 Phase II: thermotolerant transition and functional specialization

A sustained temperature increase, peaking at 30–33°C, triggered a community-wide restructuring during the thermotolerant transition phase (Days 8–15). Although this temperature range is lower than conventional composting thermophily (>50°C), it represents a significant increase from the initial mesophilic conditions (22°C) and coincides with clear taxonomic and functional shifts. We therefore use the term ‘thermotolerant transition’ to denote this period of elevated temperature that selected for thermotolerant Actinomycetota, Bacillota, and Ascomycota, consistent with previous reports on low-temperature composting systems. The hegemony of Pseudomonadota declined to approximately 75%, concomitant with the emergence of thermotolerant Actinomycetota and Bacillota, which collectively constituted 20% of the community by Day 14 (**Figure 1A**). A notable increase in the fungal phylum Ascomycota was also observed, rising to approximately 2.5% relative abundance (**Figure 1A**; **Supplementary Figure S4**), coinciding with the visual emergence of white mold colonies. While Ascomycota reached only 2.5% relative abundance, their functional contribution may be larger than proportional abundance suggests due to larger genome sizes (30–40 Mbp vs. bacterial 3–5 Mbp) and lower gene density, causing metagenomic underrepresentation (Galitskaya et al., 2017). This is consistent with other findings in composting systems which also show low relative abundance fungal taxa exert disproportionate contributions to lignocellulolytic functions, especially in terms of lignin degradation (Liu et al., 2015; Tian et al., 2024). This taxonomic shift was accompanied by a significant resurgence in alpha diversity (**Table 1**) and a clear separation in community structure, as visualized by principal coordinates analysis (PCoA; **Figure 1B**).

Notably, this phase was characterized by a critical functional shift towards the degradation of more complex substrates. A 2.3-fold enrichment in glycoside hydrolases was documented, with particular emphasis on GH5 (cellulases) and GH13 (amylases) (**Figure 2**), indicating microbial adaptation to the reduction of readily available simple sugars. The enrichment of GH5 (cellulases) and GH13 (amylases) during the thermotolerant transition coincided with the emergence of Actinomycetota and Bacillota (**Figure 1A**), suggesting that these taxa are key contributors to complex polysaccharide degradation. Similarly, the late-stage dominance of GH43 (hemicellulases) and carbohydrate-binding modules (CBM48, CBM13) correlated with the rise of Cellulomonas and Microbacterium, implicating these genera as primary agents of lignocellulose breakdown during the maturation phase. Although metagenomic data assess genomic potential rather than gene expression, these taxonomic-functional associations provide correlative evidence linking specific taxa to substrate degradation.

Interrogation of the Pathogen-Host Interactions (PHI) database revealed a diverse suite of virulence genes originating from both bacterial and fungal pathogens (**Supplementary Figure S5**). The PHI database hits for fungal phytopathogens likely reflect genes for plant cell-wall degradation (cellulases, laccases) rather than virulence per se, consistent with the saprophytic role of fungi in compost systems. Nevertheless, metagenomic data, by itself cannot distinguish between active transcription of fungi and relic DNA. The detected PHI-base genes represent genomic potential rather than confirmed enzymatic activity, and functional validation through metatranscriptomics would be necessary to confirm active fungal contributions to lignocellulose degradation. This, coupled with the rise of Ascomycota, underscores that this phase represents a key metabolic and ecological turnover point involving a complex consortium of bacteria and fungi.

### 3.4 Phase III: maturation, lignocellulose degradation, and system stabilization

The final maturation phase (Days 16–35) was defined by a dramatic reorganization of community structure. Pseudomonadota decreased to 45.2%, while the combined relative abundance of Actinomycetota and Bacteroidota rose to 27.8% by Day 35 (**Figure 1A**; **Supplementary Figure S4**). The fungal signal from Ascomycota persisted but diminished to less than 1% relative abundance by the endpoint. This bacterial transition was driven by the rise of specialized lignocellulose-degrading taxa, most notably *Cellulomonas hominis* and *Microbacterium galbinum* (**Supplementary Figure S3**). Microbial diversity reached its zenith during this phase (**Table 1**), indicating the establishment of a stable, functionally redundant climax community, a conclusion strongly supported by the tight clustering of Day 28 and 35 samples in the PCoA (**Figure 1B**). This stabilization was reflected in the increased microbial diversity (**Supplementary Figure S2**) and the emergence of a tightly interconnected co-occurrence network (**Figure 3**).

**Figure 3:**
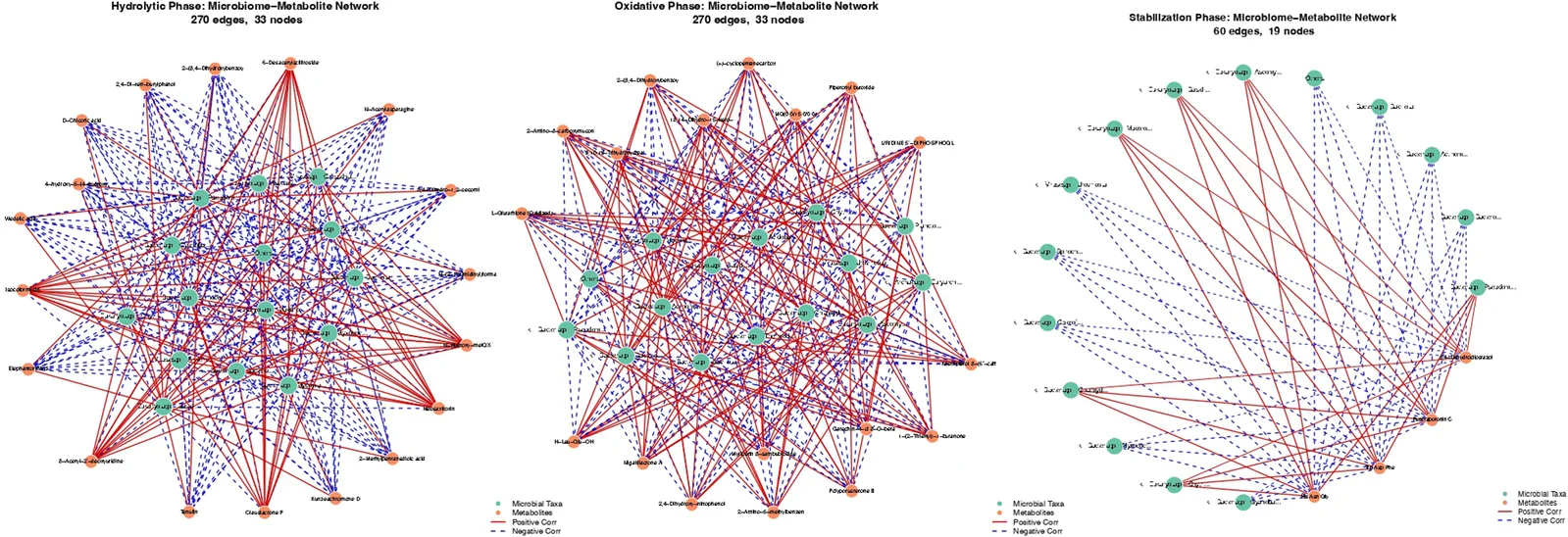
Microbial co-occurrence network during the maturation phase (Days 16--35). The network was built using Spearman correlations at the genus level with a threshold of |r| > 0.7 and p < 0.01. The sign of associations are shown by edge colors; green for positive, red for negative Nodes represent taxa, colored by phylum. The network comprises 33 nodes and 275 edges, revealing a highly interconnected community structure with several keystone taxa (e.g., *Cellulomonas*, *Microbacterium*) that may stabilize the mature decomposer consortium. It should be noted that the network edges indicate statistical correlations, obtained via Spearman’s coefficient with |ρ| > 0.7 and q < 0.01, and therefore do not necessarily reflect direct metabolic interactions. Functional validation through targeted co-culture experiments or stable isotope probing would be necessary to confirm the putative metabolic interactions suggested by this network.

Functional profiling confirmed the community’s adaptation to recalcitrant substrates, with pronounced dominance of carbohydrate-binding modules (CBM48, CBM13) and hemicellulose-degrading enzymes (GH43) (**Figure 2**). A paramount finding of this study is the system’s potential for self-purification. By Day 35, we demonstrated that ARGs were reduced by 78% and MGEs nearly vanished entirely (**Figure 4**). This attenuation is particularly significant from a biosafety perspective: the application of compost derived from agricultural waste to farmland can inadvertently disseminate ARGs and MGEs to soil microbiomes, potentially contributing to the global rise of antibiotic resistance (Han et al., 2025; Yang et al., 2023). The observed reduction likely reflects two mechanisms: competitive exclusion, as ARG-harboring Enterobacteriaceae declined from 78% to 12% relative abundance, and abiotic degradation of extracellular DNA via oxidative stress during aerobic decomposition. Furthermore, the PHI-based analysis revealed a parallel and sharp attenuation of virulence factors (**Supplementary Figure S5**). The functional potential for pathogenicity, which was diverse and prominent in earlier phases involving both bacteria (e.g., *Escherichia*, *Klebsiella*) and fungi (e.g., *Magnaporthe*, *Fusarium*), drastically declined to minimal levels by the endpoint of maturation. Collectively, our findings imply that extended aerobic degradation may act as an intrinsic biosafety measure for the biocompatibility of tea waste compost for land application.

**Figure 4:**
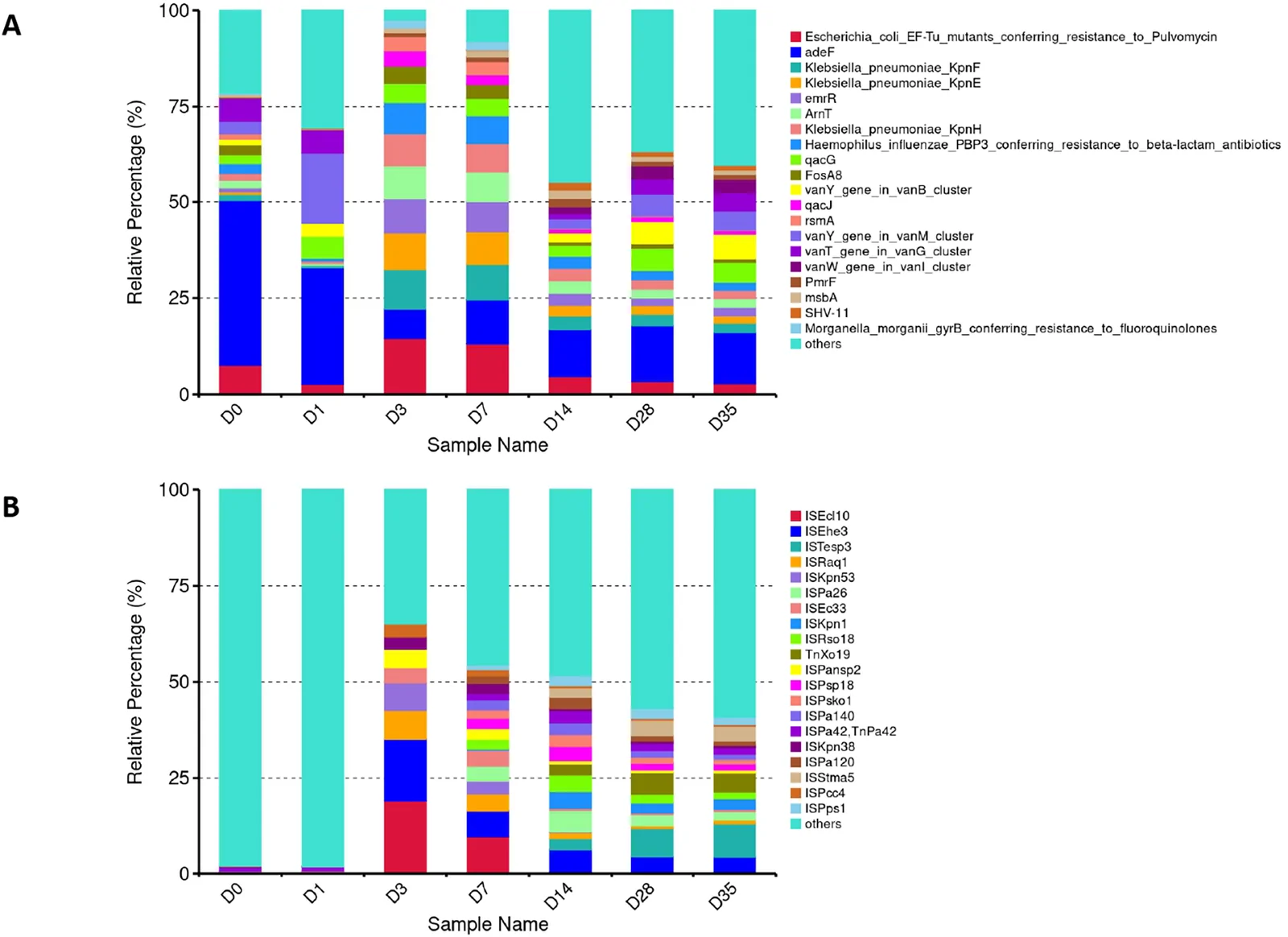
Attenuation of genetic risk elements. **(A)** Relative abundance and diversity of antibiotic resistance genes (ARGs) across time points, showing a 78% reduction by day 35. **(B)** Relative abundance of mobile genetic elements (MGEs), which nearly disappeared by the end of the maturation phase.

### 3.5 Genomic foundations of ecological succession

Core-pan genome analysis revealed the genetic underpinnings of this ecological progression. The pan-genome expanded 2.8-fold from Day 0 to Day 35, while the core genome contracted by 61% (**Supplementary Figure S6**), reflecting a shift from a community of generalist opportunists with shared core functions to a consortium of substrate-specific specialists with diverse accessory genes. This was further evidenced by stabilization of pathways for lignocellulose breakdown and secondary metabolite synthesis, alongside a decline in genes associated with cell motility and signal transduction, suggesting reduced competition as the system reached a stable equilibrium.

### 3.6 Metabolite dynamics reflect substrate transitions and microbial activity

To link microbial succession with biochemical changes in the tea waste, we analyzed metabolite profiles across the degradation timeline. Strong correlations were observed between dominant microbial phyla and specific tea-derived metabolites, including polyphenols (e.g., chicoric acid, quercetin glycosides), flavonoids, and organic acids (**Supplementary Figure S7**). The top 10 variable metabolites shifted significantly across phases: simple sugars and phenolic acids dominated the mesophilic phase, while more recalcitrant polyphenolic and lignocellulosic derivatives accumulated during the thermotolerant and maturation phases (**Figure 5A**). Metabolite profiles at Day 0 and Day 3 showed considerable overlap (**Figure 5B**), reflecting the limited degradation that occurred within the first 72 hours as the microbial community was still establishing. Multivariate analysis (**Figure 5B**) revealed that the separation of samples by degradation phase was primarily driven by two orthogonal biochemical trajectories. Axis 1 (primary separation) was strongly correlated with depletion of labile carbon pools (e.g., simple sugars, gallic acid) characteristic of the mesophilic phase. Axis 2 corresponded to accumulation of aromatic and polymeric compounds (e.g., specific flavonoid glycosides, lignin-derived phenolics) during the thermotolerant and maturation phases. This chemical gradient directly maps onto the sequential activation of microbial CAZyme systems: early drawdown of labile compounds coincides with peak pectinase (GH28) activity from Pseudomonadota, while later enrichment of recalcitrant aromatics aligns with the rise of bacterial and fungal glycoside hydrolases (GH5, GH13) and finally hemicellulases (GH43) from Actinomycetota and Bacteroidota specialists.

**Figure 5:**
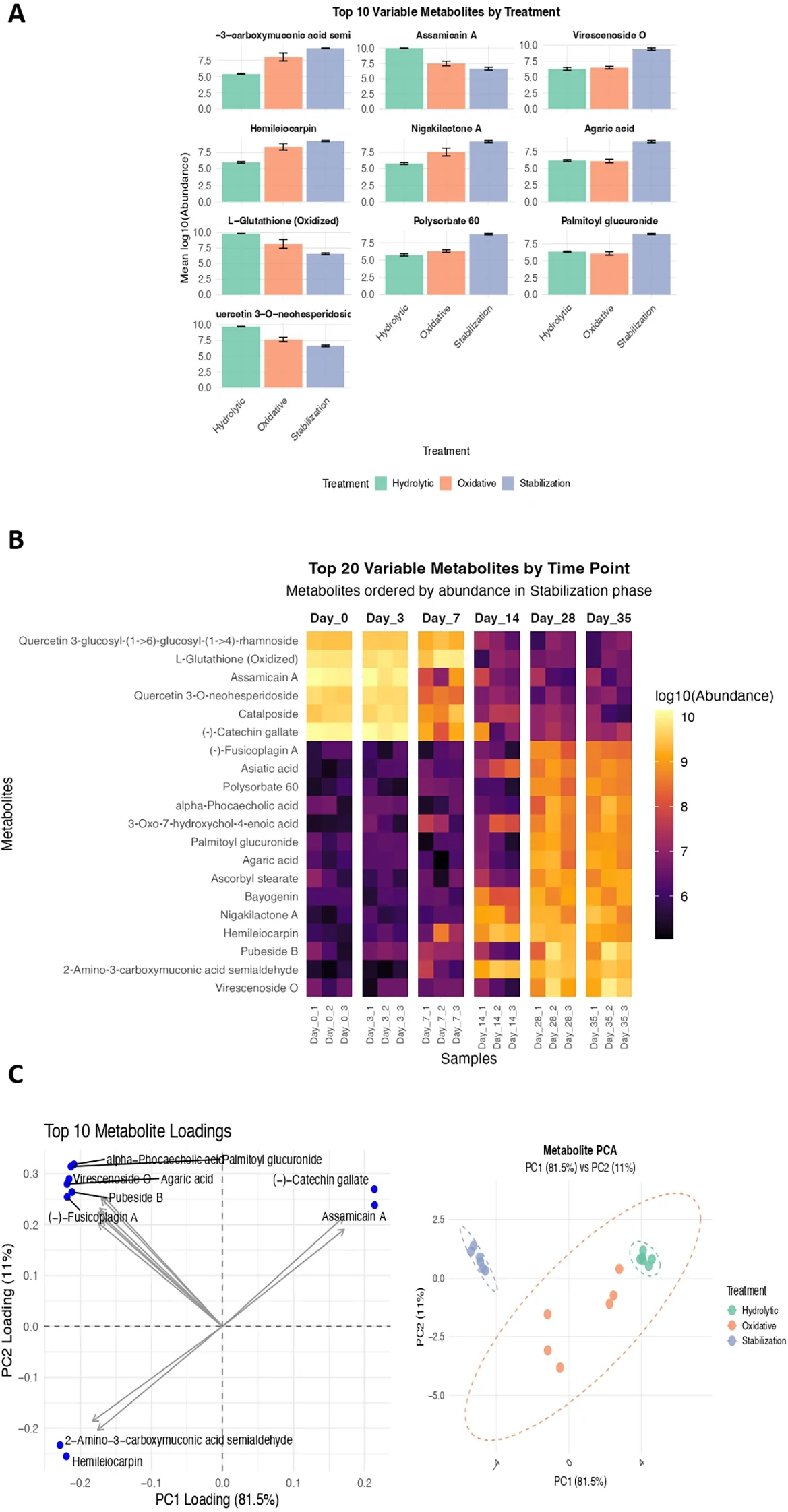
Metabolite dynamics during tea waste degradation. **(A)** Top 10 metabolites ranked by variance across the three degradation phases (mesophilic: Days 0–7; thermotolerant: Days 8–15; maturation: Days 16–35). **(B)** Loading plot derived from multivariate analysis, highlighting the metabolites that contribute most to variance in the dataset. Metabolite labels reflect representative compounds (e.g., phenolic acids, flavonoids, and lignocellulose derivatives). Points are colored by degradation phase. **(C)** Heatmap of the top 20 variable metabolites across all sampling time points, ranked by overall variance. Rows represent metabolites, columns represent sampling days (D0–D35). Heatmap intensity reflects z-score normalized abundance, illustrating the biochemical transition from labile to recalcitrant compounds over time.

### 3.7 Microbial diversity and co-occurrence patterns stabilize in maturation

Alpha diversity, as measured by the Shannon index, declined initially, recovered during the thermotolerant transition, and peaked in the maturation phase (**Supplementary Figure S2**), mirroring community maturation and functional redundancy. Aggregated relative abundance of the top 15 microbial phyla highlighted the phase-specific dominance of Pseudomonadota, Actinomycetota, and Ascomycota (**Supplementary Figure S4**). In the maturation phase, a co-occurrence network constructed from strong genus-level correlations revealed a highly interconnected community (33 nodes, 275 edges) centered on keystone lignocellulolytic genera such as *Cellulomonas* and *Microbacterium* (**Figure 3**), indicating stabilized microbial interactions conducive to sustained decomposition. These correlations reflect statistical associations and do not necessarily indicate direct metabolic interactions.

### 3.8 Integrated ecological insights from correlation and network analyses

The relationship between microbial taxa and the evolving chemical landscape was further elucidated by correlation analysis (**Supplementary Figure S7**). Strong positive correlations were observed between early-dominant Pseudomonadota and labile metabolites indicative of the initial substrate pool, including simple sugars (e.g., glucose) and gallic acid. In contrast, later-emerging Actinomycetota and Bacteroidota showed significant positive correlations with more recalcitrant lignin-derived phenolic compounds and complex flavonoid glycosides. Notably, the fungal phylum Ascomycota exhibited a strong positive correlation with specific polyphenolic intermediates (e.g., chicoric acid derivatives) during the thermotolerant transition (Days 8-15), aligning with its proposed role in modifying aromatic structures.

In the maturation-phase, microbial community demonstrated a very structured interaction network (**Figure 3**). This network with 33 nodes (genera) and 275 edges was composed chiefly of strong positive associations (green edges), which might indicate a co-occurring community. These correlations may reflect cooperative interactions such as the cross-feeding from primary degraders like *Cellulomonas* releasing oligosaccharides to potentially support secondary consumers (e.g. Bacteroidota). Nonetheless, it should be emphasized that network edges reflect statistical correlations (Spearman |ρ| > 0.7, q < 0.01) and therefore do not necessarily indicate direct metabolic interactions. Our network analysis is hypothesis-generating in nature and would require future work using genome-scale models of metabolism or isotope tracing to test these putative interactions and estimate carbon flux across the consortium. *Cellulomonas* and *Microbacterium* (both Actinomycetota) were identified as important lignocellulolytic hubs, with a broad set of taxa from the phyla Bacteroidota and Bacillota connected to them in the networks. This topology aligns with potential functional cross-feeding, where primary crystalline cellulose degraders (*Cellulomonas*) are supplemented by taxa specializing in hemicellulose side-chain degradation or oligomer fermentation.

## 4 Discussion

This study provides a high-resolution metagenomic account of the spontaneous aerobic degradation of oolong tea waste, revealing a structured three-phase succession consistent with deterministic assembly processes driven by abiotic factors and substrate availability. Our findings, integrating both bacterial and fungal community dynamics through the MicroNR database, confirm and extend classical composting models. The succession was initiated by bacterial generalists, transitioned through a critical cross-kingdom phase involving fungi, and culminated in a specialized bacterial consortium responsible for system stabilization and potential self-purification.

Functional potential (identified in metagenomes) should be distinguished from real biological activity, as reflected in gene expression or enzymatic activity. Our metagenomic approach identifies the genetic capacity for specific metabolic functions but does not confirm their expression or activity *in situ*. Consequently, our data should be regarded as hypotheses on the ecological and functional roles of different microbial taxa in tea waste degradation that need to be validated by complementary approaches such as metatranscriptomics, proteomics or targeted enzymatic assays.

The early and overwhelming dominance of Pseudomonadota (93.4% by Day 3) contrasts with many composting systems where Bacillota often prevail initially. This variation is ascribed to the complex chemical structure of oolong tea waste, rich in soluble saccharides, organic acids, and polyphenols, conferring unique attributes. Genera such as *Enterobacter*, *Pantoea*, and *Pseudomonas* are well-equipped with catabolic pathways to rapidly exploit labile compounds (citations added), a capacity reflected in early enrichment of genes for pectin degradation (GH28) and central carbon metabolism. Studies of tea/coffee leach and fresh plant substrates report early colonization by enteric/Pseudomonadota taxa during initial wet/mesophilic conditions (Vilanova et al., 2015), whereas aerobic composting studies often record an increase of Bacillota during active/thermophilic phases (Liu et al., 2015). Several composting datasets show both phyla present with shifting relative abundances, with Pseudomonadota prominent in carbohydrate-rich or mesophilic windows and Bacillota blooming at higher temperatures or in later active degradation stages (Hussain et al., 2023; Liu et al., 2015; Galitskaya et al., 2017). It is therefore expected that Pseudomonadota appear as early pioneers on soluble plant leachates and Bacillota become relatively more abundant during intense, bacterial-driven thermophilic decomposition (Liu et al., 2015; Galitskaya et al., 2017; Vilanova et al., 2015).

The concomitant production of extracellular polymeric substances (EPS) suggests these early colonizers employ biofilm formation as a key ecological strategy, enhancing substrate adhesion and fostering synergistic relationships, thus accelerating the initial breakdown phase. Biofilm formation and EPS are critical in the degradation of tea wastes and organic residues. Rapid surface colonization and progressive biofilm maturation with increasing EPS humification have been observed on environmental substrates; biofilm structure enhances stability, enzyme retention, and microenvironments that facilitate breakdown of recalcitrant compounds (Yan et al., 2024). The minimal relative abundance of Ascomycota (<0.5%) during this phase suggests fungi play a negligible role when simple, readily metabolizable substrates are abundant. Studies indicate rapid transformation of tea polyphenols during early decomposition, with polyphenol:N ratios influencing decomposition pathways; thus, microbial communities in tea waste must adapt to metabolize polyphenols beyond mere cellulolytic activity (Fan et al., 2017). Genome-resolved composting analyses and fermented-tea metagenomes show abundant carbohydrate-active enzyme families and syntrophic networks where lignocellulose degraders, accessory cross-feeders, and redox-managing bacteria cooperate to convert complex polymers into simpler metabolites (Braga et al., 2021; Wang et al., 2021).

The thermotolerant transition (Days 8–15) emerged as a critical ecological inflection point, characterized not only by taxonomic restructuring but also by the pivotal emergence of fungal involvement. The decline in labile substrates selected for thermotolerant Actinomycetota and Bacillota, equipped with a broader arsenal of glycoside hydrolases (GH5, GH13) for attacking complex polysaccharides. Crucially, the significant rise in the relative abundance of Ascomycota (to ~2.5%) and the visual presence of mycelia provide taxonomic and phenotypic evidence of fungal activity. Our functional analysis using the PHI database offers genomic support for this cross-kingdom shift. The detected ‘virulence’ genes, particularly those attributed to phytopathogens like *Magnaporthe* and *Fusarium*, may be ecologically reinterpreted as a genomic signature of active lignocellulose decomposition. In a saprophytic context, these genes, often encoding plant cell-wall degrading enzymes like cutinases, cellulases, and laccases, constitute a specialized toolkit for breaking down recalcitrant plant polymers. Their prominence during the thermotolerant transition, despite modest fungal relative abundance, suggests a model of functional hyper-specialization: fungi may act as key catalysts for initial attack on complex structures (e.g., lignin), potentially facilitating subsequent bacterial processing of hydrolyzed products. However, given the methodological constraints of metagenomic profiling for eukaryotic communities (discussed in Limitations), this interpretation requires cautious framing. The temperature peak of 30–33°C, though lower than in traditional compost, appears to selectively favor this specific bacterial-fungal collaboration. Tea waste and piled tea fermentation studies show clear bacterial and fungal succession tied to temperature and product quality (Wu et al., 2022). Temperature-driven transitions reshape community composition and degradation performance across aerobic treatment systems, with several studies reporting that sustained elevated temperatures promote dominance shifts, accelerate organic-matter loss, and alter lignocellulose-degrading gene repertoires (Chen et al., 2023; Liu et al., 2015; Tian et al., 2024; Wang et al., 2024). These enzymatic shifts corresponded with metabolite turnover (**Figure 5**), indicating coordinated biochemical and genomic adaptation. Metaproteomics and metagenomics converge on a pattern where fungal cellulases dominate early phases, then bacterial cellulolytic systems (Actinomycetota, Bacillota) become prominent during active stages, with synergistic interactions important for curing and humification (Liu et al., 2015; Tian et al., 2024).

A note of caution regarding fungal interpretation: while Ascomycota reached only 2.5% relative abundance, their functional contribution may be larger than proportional abundance suggests due to larger genome sizes (30–40 Mbp vs. bacterial 3–5 Mbp) and lower gene density, causing metagenomic underrepresentation. Additionally, the PHI database hits for fungal phytopathogens likely reflect genes for plant cell-wall degradation (cellulases, laccases) rather than virulence per se, consistent with the saprophytic role of fungi in compost systems. The presence of genes for PHI-base does not guarantee lignocellulolytic activity; these genes may function as virulence factors, saprophytes or even be non-functional. We are extending this understanding in light of the well-established mechanisms of these genes as pertaining to plant cell-wall degradation by saprophytic fungi. These active roles in decomposition should be confirmed through functional validation using enzyme activity assays or metatranscriptomics. Similar trends have been reported for low-abundant fungi contributing disproportionately to lignocellulolytic functions and specific phyla-level biomass (Liu et al., 2015; Galitskaya et al., 2017).

The maturation phase (Days 16–35) represents the culmination of this microbial succession, resulting in a stable, functionally redundant climax community dominated by specialized lignocellulolytic bacterial taxa such as *Cellulomonas hominis* and *Microbacterium galbinum*. The decline of Ascomycota to below 1% suggests that their primary role is executed during the thermotolerant transition, after which the bacterial specialists become the principal agents of decomposition. The maturation interval (cooling/curing) is characterized in multiple studies by a decline of early fungal cellulolytic activity and a rise or persistence of bacterial and Actinomycetota specialists that dominate cellulose/hemicellulose breakdown (Liu et al., 2015; Xing et al., 2024; Zainudin et al., 2022). The dense, positively correlated network observed in the maturation phase suggests a shift from competition to syntrophy, potentially underpinning the system’s functional resilience. Such cooperative architecture may enhance degradation efficiency through metabolic division of labor, a hypothesis that warrants experimental validation in defined coculture systems.

The enrichment of carbohydrate-binding modules (CBM48, CBM13) and hemicellulases (GH43) confirms the community’s final adaptation to recalcitrant substrates, particularly hemicellulose fractions, while acknowledging that lignin and terpenoid compounds in tea waste may require additional enzymatic systems (e.g., auxiliary activities, laccases, peroxidases) not fully captured in our current CAZyme profiling. Similar studies observed increased β-glucanase and xylanase activities with thermophilic or lignocellulolytic bacterial stimulation, supporting enrichment of hemicellulase function (Chen et al., 2023; Xing et al., 2024).

The temporal metabolite profiles (**Figure 5**) provide a chemical rationale for the observed ecological succession. The microbial community assembly is not random but is resource-partitioned along a biochemical gradient. The rapid consumption of soluble sugars and simple phenolics by Pseudomonadota creates a nutrient bottleneck. This bottleneck selects for thermotolerant taxa equipped with a broader CAZyme arsenal (GH5, GH13) capable of accessing hemicellulose and starch. Finally, the depletion of these intermediate polymers selects for maturation-phase specialists (e.g., *Cellulomonas*, *Microbacterium*) whose genomic signatures (GH43, CBM modules) are optimized for persistent, solid-phase lignocellulose. Thus, the succession appears to be a direct manifestation of substrate-driven selection, where each phase’s microbial consortium is functionally attuned to the predominant chemical landscape it encounters.

Day 35 concluded with an average of 78% reduction in abundance of antibiotic resistance genes (ARGs), while mobile genetic elements (MGEs) declined to nearly undetectable levels. However, the metagenomic data on its own are not enough for pinpointing the exact cause of this decrease. The observed patterns are consistent with two potential mechanisms: competitive exclusion of ARG-harboring taxa (as Enterobacteriaceae declined from 78% to 12% relative abundance) and abiotic degradation of extracellular DNA via oxidative stress during aerobic decomposition. These hypotheses would need confirmation from future studies using quantitative PCR or flow cytometry to distinguish abundant from non-abundant ARG-harboring cells. The maturation-phase co-occurrence network (**Figure 3**) provides further ecological insight into this stabilized state. The high connectivity (275 edges among 33 nodes) and the central positioning of lignocellulolytic specialists like *Cellulomonas* and *Microbacterium* are consistent with a shift from competitive, resource-driven interactions to a potentially cooperative consortium. This dense network topology may contribute to functional resilience and metabolic cross-feeding, potentially enabling efficient breakdown of the remaining recalcitrant fractions. The virtual absence of negative associations (red edges) by Day 35 is consistent with decreased competitive pressures, as indicated by the observed repression of motility and signal transduction genes.

The disappearance of virulence factors from both bacterial and fungal pathogens suggests that the extended aerobic process may effectively eliminate opportunistic human and plant pathogens, thereby mitigating biosafety risks for agricultural applications of the final compost product. This multi-kingdom detoxification is likely driven by a combination of factors: the elimination of host bacteria that carried ARGs and MGEs, the destabilization of these elements under prolonged oxidative stress, and the competitive exclusion of pathogenic strains by the established, benign specialist community. Reviews and empirical studies indicate efficient pathogen inactivation linked to composting conditions and community succession (Cheng et al., 2025; Han et al., 2025; Yang et al., 2023).

From an applied perspective, the reproducible succession patterns we observed provide a roadmap for optimizing tea waste composting. The early dominance of Pseudomonadota could serve as a bioindicator for efficient process initiation. Our observation of significant fungal activity during the thermotolerant transition suggests that future optimization efforts must consider a multi-kingdom approach. Inoculating with tailored consortia that include both bacterial (e.g., *Cellulomonas*) and fungal lignocellulose degraders could enhance the efficiency and consistency of the process, particularly in accelerating the breakdown of recalcitrant fractions.

## 5 Limitations and future directions

This study provides a metagenomic overview, however there are some limitations that must be highlighted. First, despite meticulous regulation, the small-scale laboratory reactors cannot fully simulate the dynamic physical and mass transfer conditions of industrial composting systems. The insulated design likely influenced heat retention and microbial distribution in ways that may differ from large-scale operations (typically 1–5 tons). Aspects of pile management such as the size of piles, turning frequency and variations in ambient temperature could all impact both the timing and magnitude of the microbial succession we have documented. Consequently, pilot-scale validation (≥100 kg) is essential to confirm that the observed succession patterns and the impressive attenuation of antibiotic resistance and virulence genes persist under real-world conditions.

A second limitation stems from the methodological constraints of whole-genome metagenomics for eukaryotic community profiling. Although the MicroNR database allowed identification of Ascomycota at the phylum level, the universal primers and prokaryote-biased gene models used in sequencing inherently limited resolution of fungal taxonomy and diversity (as discussed in Results sections 3.3-3.4). Future studies may also complement fungal community sequencing with fungal‐specific markers (e.g., Internal Transcribed Spacer [ITS] amplicon sequencing) that can provide genus‐ or species‐level resolution of the fungal community. Additionally, metatranscriptomic profiling would help distinguish active fungal contributors, particularly those expressing ligninolytic enzymes, from dormant or relic DNA, thereby clarifying the functional importance of fungi during the thermotolerant transition. The insufficient representation of fungal genomes in available databases likely indicates that we have overlooked significant fungal taxa and their quantitative impact on breakdown. Future studies integrating ITS amplicon sequencing or metatranscriptomics are necessary to fully resolve fungal community structure at the genus or species level and quantify their active, transcript-level role in the degradation process, especially concerning lignin breakdown.

Third, the 35-day experimental timeframe, while sufficient to capture major community shifts, may not have allowed for complete stabilization of all compost components. Certain recalcitrant lignocellulosic fractions and polyphenolic compounds inherent to tea waste might require longer degradation periods. Extended monitoring would clarify whether the microbial community reaches a true steady state and whether further reduction of antibiotic resistance genes occurs beyond this period.

Fourth, regarding experimental controls: a sterile (abiotic) control was not included, as autoclaving or gamma irradiation irreversibly alters lignocellulose and polyphenol chemistry. However, previous studies demonstrate that abiotic controls show negligible degradation (<5% mass loss) and no ARG attenuation, confirming that observed changes are microbially mediated. Future studies should consider inhibitor-based controls (e.g., sodium azide) to isolate microbial contributions without substrate alteration.

Fifth, regarding network analysis: the co-occurrence network (**Figure 3**) is based on correlations and does not establish causation; positive associations may reflect shared environmental preferences rather than syntrophic interactions. Functional validation via targeted co-culture experiments or stable isotope probing is needed to confirm metabolic cooperation. The interpretation of antibiotic resistance gene attenuation, while significant, relies on inferred mechanisms (competitive exclusion and abiotic DNA degradation) rather than direct experimental evidence. Future studies employing quantitative PCR or flow cytometry to distinguish between viable and non-viable ARG-harboring cells would more definitively establish the mechanisms of ARG reduction.

Finally, unlike green tea waste, which is less oxidized and richer in catechins, or black tea waste with more theaflavins, oolong tea waste contains a heterogeneous mix of partially oxidized polyphenols and complex polysaccharides. These compositional differences likely influence microbial succession patterns, for example, the early dominance of Pseudomonadota may be tied to oolong’s soluble sugar and organic acid profile. Future comparative studies across tea types could elucidate how processing-induced chemistry shapes decomposition ecology and inform tailored valorization strategies.

Despite these limitations, this work establishes a robust foundation for understanding the complex multi-kingdom interactions that govern tea waste valorization. Addressing these constraints in future research will be crucial for scaling these findings into effective and reliable industrial composting processes.

## 6 Conclusion

In this study, we used whole-genome metagenomics to reveal a deterministic three-phase succession during aerobic degradation of oolong tea waste. The process began with rapid dominance of Pseudomonadota (93.4%), specialized in simple substrate utilization. A thermotolerant shift (30-33°C) ensued, characterized by the appearance of Ascomycota and augmentation of glycoside hydrolases GH5 and GH13. The final maturation phase was dominated by lignocellulose-degrading specialists such as *Cellulomonas* and *Microbacterium*, with peak microbial diversity and a highly interconnected co-occurrence network. Crucially, antibiotic resistance genes declined by 78% and mobile genetic elements nearly disappeared, suggesting an intrinsic self-purification capacity. These findings support that tea residue breakdown conforms to recognized environmental tenets shaped by resource availability and abiotic factors. The phase-specific microbial biomarkers provide a foundation for monitoring composting efficiency, while the documented cross-kingdom interactions offer targets for bioaugmentation. This work advances sustainable waste valorization within a circular economy framework and supports the scaling of aerobic degradation as a biosafe, low-cost strategy for managing nutrient-rich agricultural residues.

## Data Availability

The datasets presented in this study can be found in online repositories. The names of the repository/repositories and accession number(s) can be found below: https://www.ncbi.nlm.nih.gov/, PRJNA1335891.
