# Supplementary material for: Structured succession of a cross-kingdom microbiome drives degradation and detoxification of oolong tea waste
Source: Front Microbiomes. 2026 Sep 24;5:1871476. doi: 10.3389/frmbi.2026.1871476 (PMC13647172; doi:10.3389/frmbi.2026.1871476)

Supplementary Figure S1: Experimental Design and Workflow

Aerobic Degradation of Oolong Tea Waste

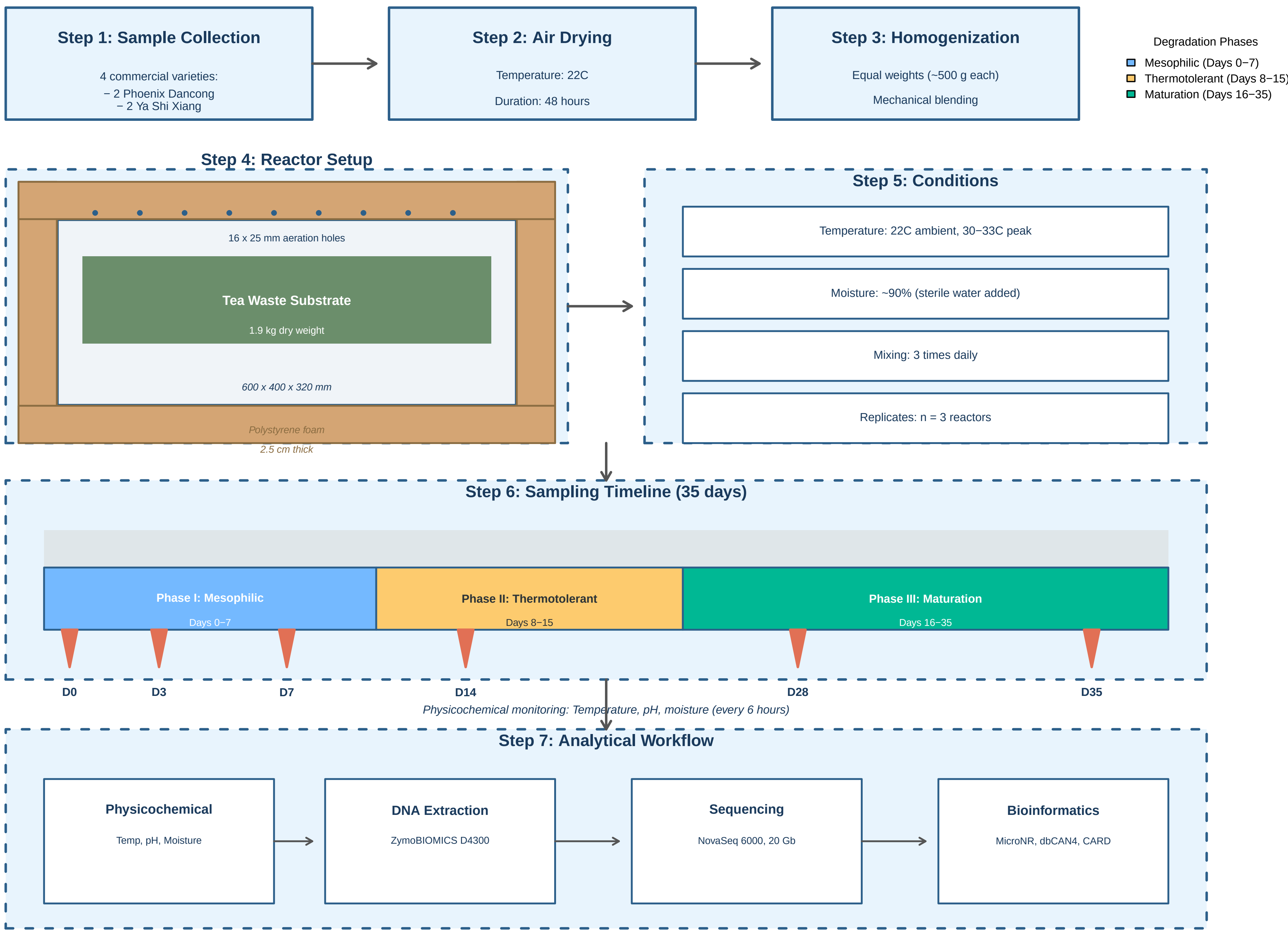

Supplement: Supplementary file 1 [file Image1.pdf]
