# Supplementary material for: Structured succession of a cross-kingdom microbiome drives degradation and detoxification of oolong tea waste
Source: Front Microbiomes. 2026 Sep 24;5:1871476. doi: 10.3389/frmbi.2026.1871476 (PMC13647172; doi:10.3389/frmbi.2026.1871476)

Supplementary Figure S2: Phase−Aggregated Alpha Diversity Indices

Aerobic Degradation of Oolong Tea Waste

Phase I: Mesophilic (Days 0−7) | Phase II: Thermotolerant (Days 8−15) | Phase III: Maturation (Days 16−35)

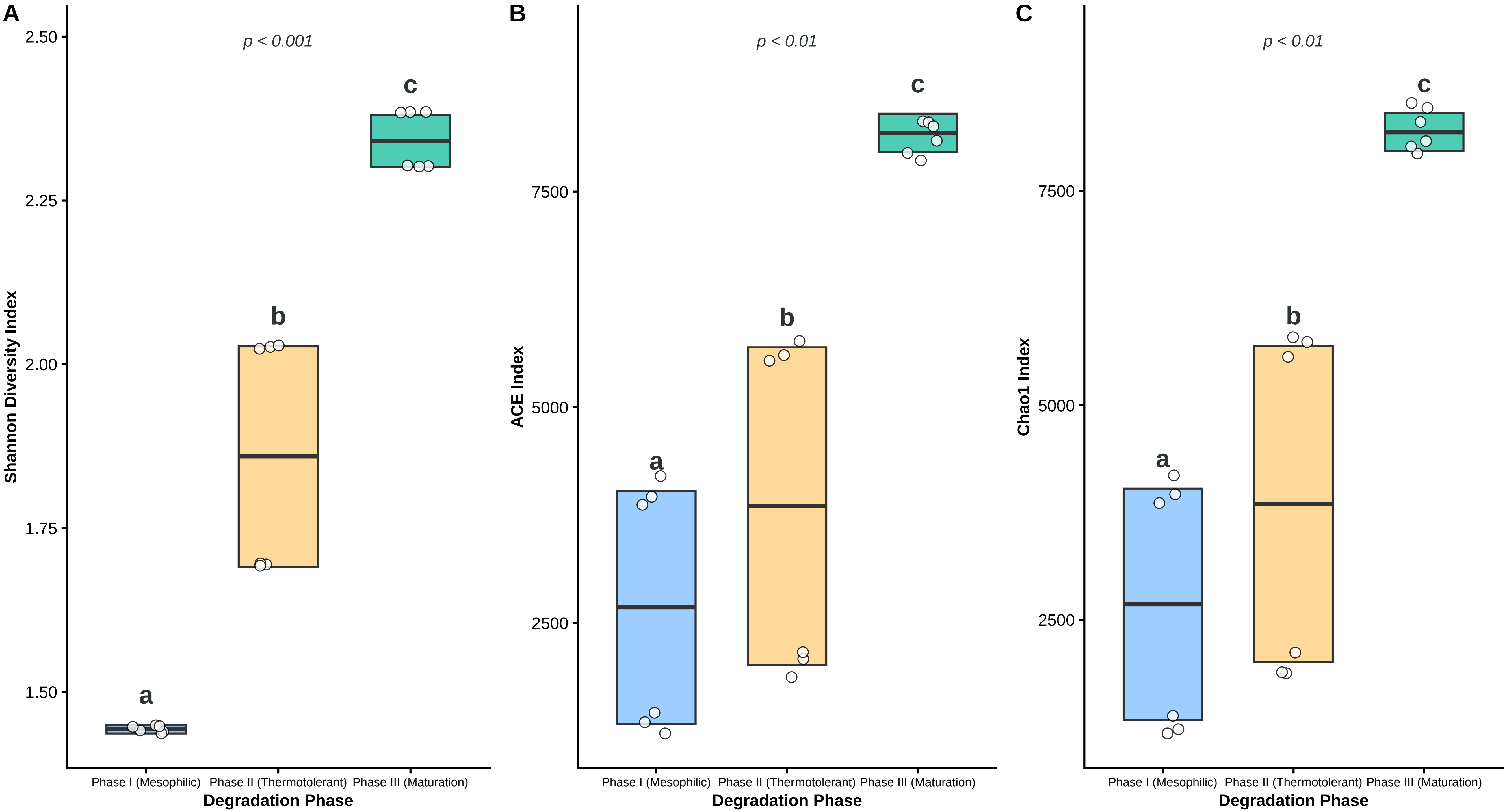

Supplement: Supplementary file 2 [file Image2.pdf]
