# Supplementary material for: Structured succession of a cross-kingdom microbiome drives degradation and detoxification of oolong tea waste
Source: Front Microbiomes. 2026 Sep 24;5:1871476. doi: 10.3389/frmbi.2026.1871476 (PMC13647172; doi:10.3389/frmbi.2026.1871476)
Supplement: Supplementary file 4 [file Image4.pdf]

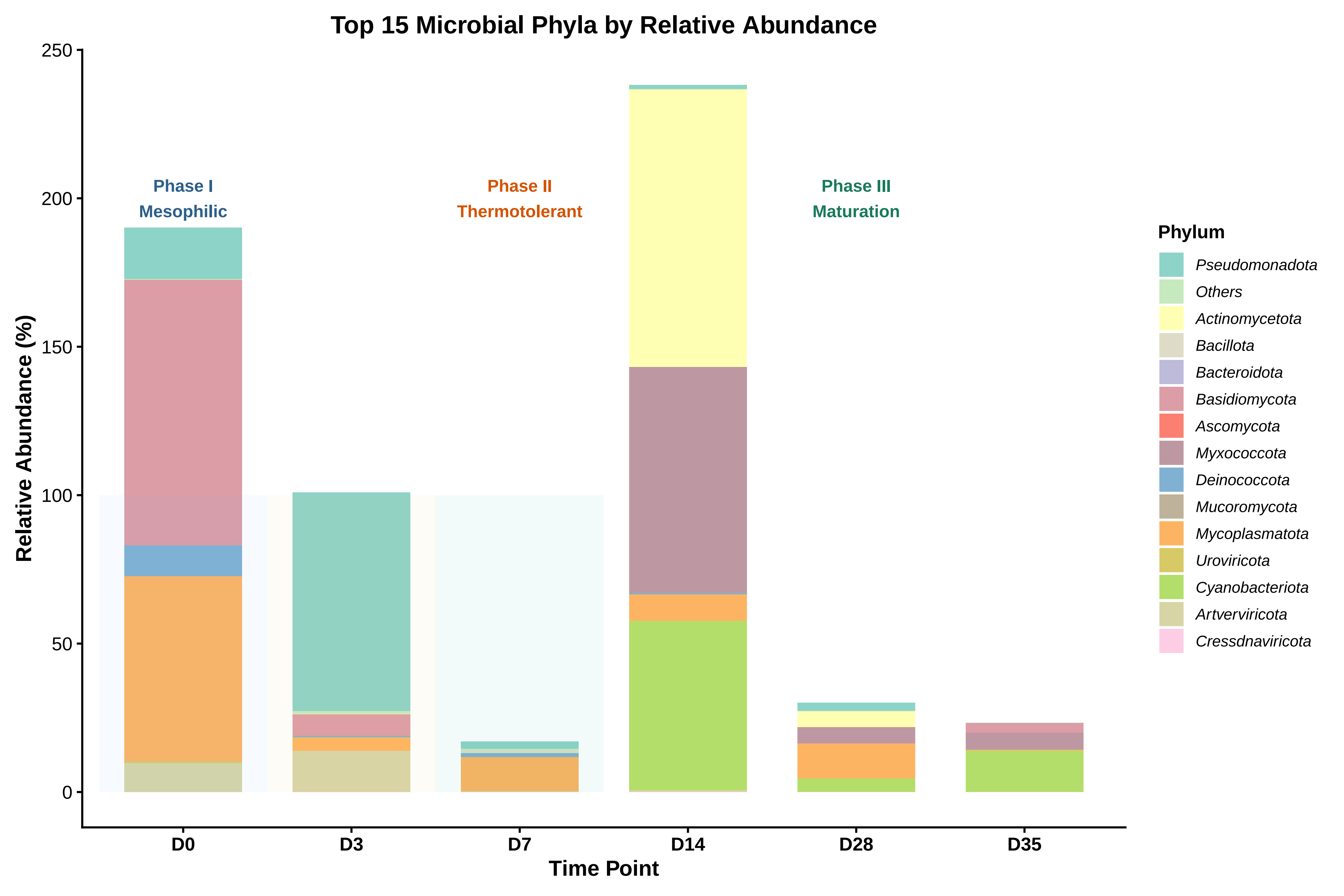
Top 15 Microbial Phyla by Relative Abundance
Relative Abundance (%)
250
200
150
100
50
0
Phase I
Mesophilic
Phase II
Thermotolerant
Phase III
Maturation
D0
D3
D7
D14
D28
D35
Time Point
Phylum
Pseudomonadota
Others
Actinomycetota
Bacillota
Bacteroidota
Basidiomycota
Ascomycota
Myxococcota
Deinococcota
Mucoromycota
Mycoplasmatota
Uroviricota
Cyanobacteriota
Artverviricota
Cressdnaviricota
