# Supplementary material for: Structured succession of a cross-kingdom microbiome drives degradation and detoxification of oolong tea waste
Source: Front Microbiomes. 2026 Sep 24;5:1871476. doi: 10.3389/frmbi.2026.1871476 (PMC13647172; doi:10.3389/frmbi.2026.1871476)

Supplementary Figure S5: PHI Virulence Factors

Aerobic Degradation of Oolong Tea Waste

Samples: D0 –> D3 –> D7 –> D28 –> D35

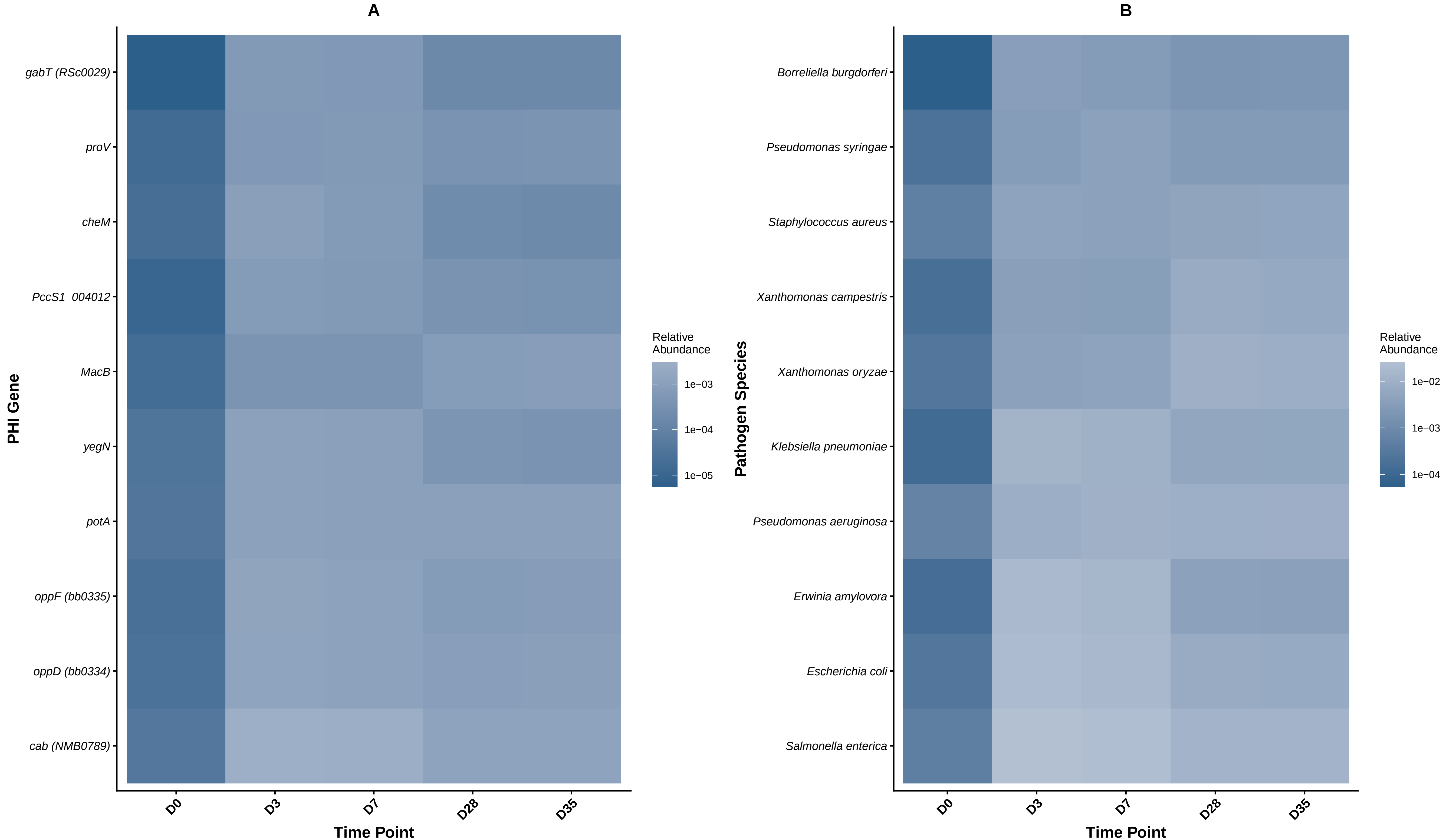

Supplement: Supplementary file 5 [file Image5.pdf]
