# Supplementary material for: Structured succession of a cross-kingdom microbiome drives degradation and detoxification of oolong tea waste
Source: Front Microbiomes. 2026 Sep 24;5:1871476. doi: 10.3389/frmbi.2026.1871476 (PMC13647172; doi:10.3389/frmbi.2026.1871476)

Supplementary Figure S6: Core−Pan Genome Dynamics

Aerobic Degradation of Oolong Tea Waste

Pan−genome expanded 2.5−fold | Core genome contracted 11%

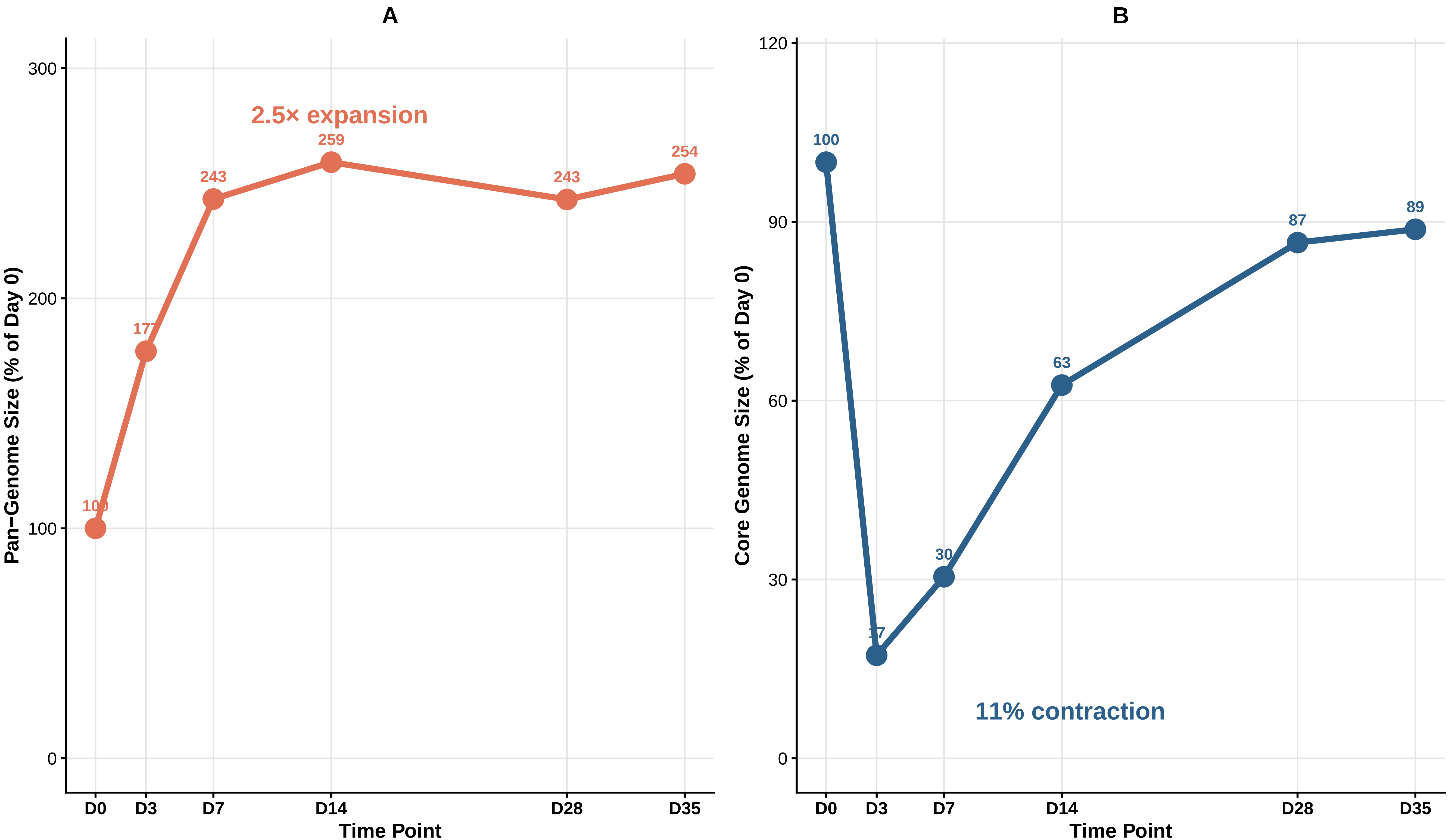

Supplement: Supplementary file 6 [file Image6.pdf]
