# Supplementary material for: Structured succession of a cross-kingdom microbiome drives degradation and detoxification of oolong tea waste
Source: Front Microbiomes. 2026 Sep 24;5:1871476. doi: 10.3389/frmbi.2026.1871476 (PMC13647172; doi:10.3389/frmbi.2026.1871476)
Supplement: Supplementary file 7 [file Image7.pdf]

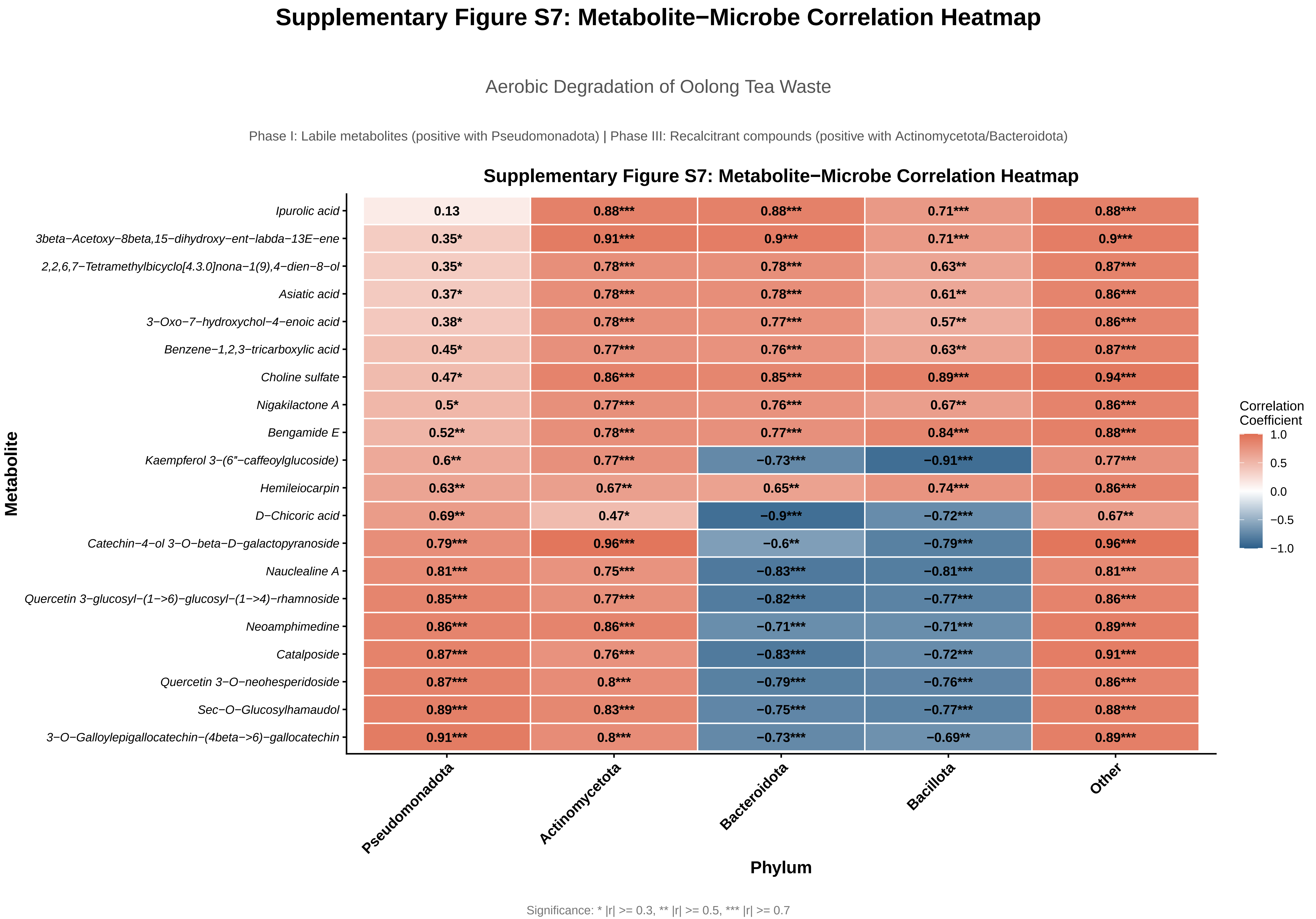

Supplementary Figure S7: Metabolite−Microbe Correlation Heatmap
Aerobic Degradation of Oolong Tea Waste
Phase I: Labile metabolites (positive with Pseudomonadota) | Phase III: Recalcitrant compounds (positive with Actinomycetota/Bacteroidota)
Supplementary Figure S7: Metabolite−Microbe Correlation Heatmap
Metabolite
Phylum
Pseudomonadota
Actinomycetota
Bacteroidota
Bacillota
Other
Ipurolic acid 0.13 0.88*** 0.88*** 0.71*** 0.88***
3beta−Acetoxy−8beta,15−dihydroxy−ent−labda−13E−ene 0.35* 0.91*** 0.9*** 0.71*** 0.9***
2,2,6,7−Tetramethylbicyclo[4.3.0]nona−1(9),4−dien−8−ol 0.35* 0.78*** 0.78*** 0.63** 0.87***
Asiatic acid 0.37* 0.78*** 0.78*** 0.61** 0.86***
3−Oxo−7−hydroxychol−4−enoic acid 0.38* 0.78*** 0.77*** 0.57** 0.86***
Benzene−1,2,3−tricarboxylic acid 0.45* 0.77*** 0.76*** 0.63** 0.87***
Choline sulfate 0.47* 0.86*** 0.85*** 0.89*** 0.94***
Nigakilactone A 0.5* 0.77*** 0.76*** 0.67** 0.86***
Bengamide E 0.52** 0.78*** 0.77*** 0.84*** 0.88***
Kaempferol 3−(6″−caffeoylglucoside) 0.6** 0.77*** −0.73*** −0.91*** 0.77***
Hemileiocarpin 0.63** 0.67** 0.65** 0.74*** 0.86***
D−Chicoric acid 0.69** 0.47* −0.9*** −0.72*** 0.67**
Catechin−4−ol 3−O−beta−D−galactopyranoside 0.79*** 0.96*** −0.6** −0.79*** 0.96***
Nauclealine A 0.81*** 0.75*** −0.83*** −0.81*** 0.81***
Quercetin 3−glucosyl−(1−>6)−glucosyl−(1−>4)−rhamnoside 0.85*** 0.77*** −0.82*** −0.77*** 0.86***
Neoamphimedine 0.86*** 0.86*** −0.71*** −0.71*** 0.89***
Catalposide 0.87*** 0.76*** −0.83*** −0.72*** 0.91***
Quercetin 3−O−neohesperidoside 0.87*** 0.8*** −0.79*** −0.76*** 0.86***
Sec−O−Glucosylhamaudol 0.89*** 0.83*** −0.75*** −0.77*** 0.88***
3−O−Galloylepigallocatechin−(4beta−>6)−gallocatechin 0.91*** 0.8*** −0.73*** −0.69** 0.89***
Correlation Coefficient
1.0
0.5
0.0
−0.5
−1.0
Significance: * |r| >= 0.3, ** |r| >= 0.5, *** |r| >= 0.7
